# Group III mGlu Receptor Agonist, ACPT-I, Exerts Potential Neuroprotective Effects In Vitro and In Vivo

**DOI:** 10.1007/s12640-013-9455-7

**Published:** 2014-01-09

**Authors:** Helena Domin, Krystyna Gołembiowska, Danuta Jantas, Katarzyna Kamińska, Barbara Zięba, Maria Śmiałowska

**Affiliations:** 1 Department of Neurobiology, Institute of Pharmacology, Polish Academy of Sciences, Smętna 12, 31-343 Kraków, Poland; 2 Department of Pharmacology, Institute of Pharmacology, Polish Academy of Sciences, Smętna 12, 31-343 Kraków, Poland; 3 Department of Experimental Neuroendocrinology, Institute of Pharmacology, Polish Academy of Sciences, Smętna 12, 31-343 Kraków, Poland

**Keywords:** ACPT-I, Neuroprotection, Group III mGlu receptors, Kainic acid, Excitotoxicity, Glutamate release

## Abstract

Many evidence suggest that metabotropic glutamate receptors (mGluRs) may modulate glutamatergic transmission, hence, these receptors are regarded as potential targets for neuroprotective drugs. Since group III mGlu receptor agonists are known to reduce glutamatergic transmission by inhibiting glutamate release, we decided to investigate the neuroprotective potential of the group III mGlu receptor agonist, (1*S*,3*R*,4*S*)-1-aminocyclopentane-1,2,4-tricarboxylic acid (ACPT-I) against kainate (KA)-induced excitotoxicity in vitro and in vivo. In primary neuronal cell cultures ACPT-I (1–200 μM), applied 30 min–3 h after starting the exposure to KA (150 μM), significantly attenuated the KA-induced LDH release, increased cell viability, and inhibited caspase-3 activity both in cortical and hippocampal cell cultures. The effects were dose-, time- and structure-dependent. The neuroprotective effects of ACPT-I were reversed by (*RS*)-alpha-cyclopropyl-4-phosphonophenyl glycine, a group III mGluR antagonist. In the in vivo studies, KA (2.5 nmol/1 μl) was unilaterally injected into the rat dorsal CA1 hippocampal region and the size of degeneration was examined by stereological counting of surviving neurons in the CA pyramidal layer. It was found that ACPT-I (7.5 or 15 nmol/1 μl), injected into the dorsal hippocampus 30 min, 1 or 3 h after KA in dose-dependent manner prevented the KA-induced neuronal damage. Moreover, in vivo microdialysis studies in the rat hippocampus showed that ACPT-I (200 μM) given simultaneously with KA (50 μM) significantly diminished the KA-induced glutamate release in the hippocampus. This mechanism seems to play a role in mediating the neuroprotective effect of ACPT-I.

## Introduction

It is generally assumed that inhibition of the toxic glutamatergic hyperactivity may be neuroprotective. Although animal studies have demonstrated neuroprotective effects of the antagonists of ionotropic glutamate receptors (iGluRs) in central nervous system injury, the results of clinical trials were unsuccessful due to adverse effects, such as ataxia, sedation, psychotic effects, and memory impairment (Muir and Lees 1995; Danysz and Parsons 1998; Ikonomidou and Turski 2002), rendering them useless in the clinic. Therefore, it seems that modulation of glutamatergic transmission may be a more promising strategy of neuroprotection than direct antagonism of the iGluRs (Lea and Faden 2003; Byrnes et al. 2009). Such indirect modulation can be achieved by compounds acting on metabotropic glutamate receptors (mGluRs). The mGluRs are classified into three groups (I–III) based on their sequence homology, pharmacological profile, and transduction pathway (Pin and Duvoisin 1995). Group I mGluRs (mGlu1 and mGlu5) are coupled to phospholipase C via Gq and their activation leads to phosphoinositide hydrolysis and intracellular mobilization of Ca^2+^ ions. Receptors of group II (mGlu2 and mGlu3) and group III (mGlu4, mGlu6, mGlu7, and mGlu8) are negatively coupled to adenylyl cyclase, and their activation inhibits the stimulated cAMP formation (Conn and Pin 1997; Spooren et al. 2003).

Group III mGlu receptors are localized predominantly on presynaptic terminals of glutamatergic and GABAergic neurons, where they are involved in the regulation of synaptic transmission (Conn and Pin 1997). It has been shown that activation of presynaptic mGlu receptors located on the glutamatergic nerve terminals causes a decrease in glutamate release, thus inhibiting excitatory glutamatergic transmission (Cartmell and Schoepp 2000; Schoepp 2001). As such, it has been suggested that the activation of these receptors may have neuroprotective effects. Indeed, a number of data have confirmed the neuroprotective properties of group III mGluR agonists in different animal models in vitro (Bruno et al. 1996, 2000; Faden et al. 1997; Gasparini et al. 1999; Lafon-Cazal et al. 1999; Iacovelli et al. 2002) and in vivo (Gasparini et al. 1999; Bruno et al. 2000; Folbergrová et al. 2008). Moreover, there are several studies showing anticonvulsant effects of group III mGluRs agonists (Chapman et al. 1999; Gasparini et al. 1999; Moldrich et al. 2001; Folbergrová et al. 2003, 2008). Therefore, these data provide evidence that group III mGluRs may be attractive targets for neuroprotective and anticonvulsive therapy.

The interesting agonist of group III mGluRs (1*S*,3*R*,4*S*)-1-aminocyclopentane-1,2,4-tricarboxylic acid (ACPT-I), with comparable potency at mGlu4 and mGlu8 receptors (Acher et al. 1997; De Colle et al. 2000; Schann et al. 2006) showed not only a potent anticonvulsant action against sound-induced seizures in rodents (Chapman et al. 2001; Moldrich et al. 2003), but also enhanced the anticonvulsant activity of AMPA or NMDA receptor antagonists (De Sarro et al. 2002). However, the neuroprotective effects of ACPT-I have not been evaluated so far.

Till date, there have been some studies showing that ACPT-I produced anxiolytic- and antidepressant-like effects after central administration (Tatarczyńska et al. 2002; Pałucha et al. 2004; Stachowicz et al. 2006; Kłak et al. 2007), and anxiolytic and antipsychotic effects after peripheral administration in animal tests and models (Pałucha-Poniewiera et al. 2008; Stachowicz et al. 2009). Furthermore, ACPT-I elicited antiparkinsonian action in animal models of Parkinson’s disease (Konieczny et al. 2007; Lopez et al. 2007; 2008; 2012) and reduced neuropathic or inflammatory hyperalgesia in animal models of pain (Goudet et al. 2008).

It should be noted that in the majority of studies reporting the neuroprotective effects of group III mGluR agonists, the drugs were applied predominantly before, simultaneously or shortly after the damage. Such procedures are greatly different from the situation that may be faced in clinical practice, where a therapeutic drug must exert its neuroprotective effect when given after the insult. Therefore, in the present study, we tried to determine whether ACPT-I produces neuroprotective effects against excitotoxicity induced by kainic acid (KA) in primary cortical and hippocampal cultures of mouse neurons and after intrahippocampal injections in rats, particularly after delayed treatment. The model of KA-induced neurodegeneration was chosen as a good and validated simulation of various pathological effects of toxic glutamatergic overactivation that occurs, for example, in epilepsy, ischemia, and traumatic injures (Coyle 1983; Ferkany and Coyle 1983; Wang et al. 2005), because KA acts not only directly via postsynaptic KA receptor stimulation but also by the secondary massive release of endogenous glutamate which activates all the glu receptors and leads to neurodegeneration (Ferkany et al. 1982; Ferkany and Coyle 1983). Moreover, the KA-induced neurodegeneration develops slowly, which makes it useful for studies of the delayed neuroprotection (Mazzone et al. 2010; Mazzone and Nistri 2011). Therefore, in the present study, both in vitro and in vivo, we assessed the effectiveness of ACPT-I in protecting neurons against KA toxic effects, even if neuroprotectant was applied 30 min, 1, 3, or 6 h after the toxic insult. In addition, in order to examine whether the mechanism of action of ACPT-I may be related to the reduction of excitatory glutamatergic transmission, microdialysis experiments were conducted in the hippocampus of freely moving rats.

## Materials and Methods

### In Vitro Studies

#### Chemicals

(1*S*,3*R*,4*S*)-1-aminocyclo-pentane-1,3,-4-tricarboxylic acid (ACPT-I; Tocris, USA), (*RS*)-alpha-cyclopropyl-4-phosphonophenylglycine (CPPG; Tocris, USA), and KA (Tocris, USA). Neurobasal A medium and supplement B27 were from Gibco (Invitrogen, Poisley, UK). The Cytotoxicity Detection Kit was from Roche Diagnostic (Mannheim, Germany). All other reagents were from Sigma-Aldrich (St. Louis, MO, USA).

#### Primary Neuronal Cell Cultures

The experiments were conducted on primary cultures of a mouse cortical and hippocampal neurons. All the procedures were carried out in accordance with the Local Bioethical Commission Guide for the Care and Use of Laboratory Animals. Neuronal tissues were taken from Swiss mouse embryos at 15–17 days of gestation and were cultivated essentially as described previously (Brewer 1995; Kajta et al. 2007). Pregnant females were anesthetized with CO_2_ vapor, killed by cervical dislocation, and subjected to cesarean section in order to remove the fetal brains. The dissected cortical and hippocampal tissues were separately minced into small pieces, then digested with trypsin [0.1 % for 15 min at room temperature (RT)], triturated in the presence of 10 % fetal bovine serum (FBS) and DNAse I (170 Kunitz units/ml), and finally centrifuged for 5 min at 1,000×*g*. The cells were then suspended in Neurobasal medium supplemented with 5 % FBS and plated at a density of 1.5 × 10^5^ cells per cm^2^ onto poly-ornithine (0.01 mg per ml)-coated multi-well plates (TPP). After 2 days, the culture medium was exchanged to Neurobasal medium supplemented with B27 (200 μl/100 ml). This procedure typically yields cultures that contain about 90 % neurons and 10 % astrocytes (Kajta et al. 2004). The cultures were maintained at 37 °C in a humidified atmosphere containing 5 % CO_2_ for 8 days prior to the experimentation.

#### Treatment

In order to evoke toxic effects, primary neuronal cultures were exposed to KA (150 μM) for 24 h (hippocampal cultures) or 48 h (cortical cultures). The concentration of KA used in our experiments as well as the end points for particular measurements (LDH release and caspase-3 activity) were chosen on the basis of our earlier studies (Domin et al. 2006; Śmiałowska et al. 2009). In order to prevent the toxic and apoptotic effects of KA, the cell cultures were treated with group III mGlu receptor agonist, ACPT-I (1, 10, 100, or 200 μM) 30 min, 1, 3, or 6 h after starting the exposure to KA. The concentrations of ACPT-I were chosen on the basis of our pilot experiments. In addition, a potent and selective group III mGlu receptor antagonist, CPPG (20, 100 or 200 μM) was applied 10 min before the ACPT-I. The concentrations of CPPG were chosen due to the findings of Toms et al. (1996) and Evans et al. (2000). All the compounds were dissolved in redistilled water and were present in cultures at a final concentration of 0.1 %. The control cultures were treated with the same amount of the respective solvent.

#### Measurement of Lactate Dehydrogenase (LDH) Activity

In order to quantify cell death, lactate dehydrogenase (LDH) released from damaged cells into the cell culture media was measured 24 h (hippocampal cultures) and 48 h (cortical cultures) after treatment with KA. A colorimetric assay was applied, according to which the amount of a formazan salt, itself formed by the conversion of lactate to pyruvate and then by the reduction of tetrazolium salt, was proportional to LDH activity in the sample. Cell-free culture supernatants were collected from each well and incubated with the appropriate reagent mixture according to the supplier’s instructions (Cytotoxicity Detection Kit, Roche) at RT for 20 min. The intensity of the red color formed in the assay and measured at a wavelength of 490 nm was proportional to the LDH activity and to the number of damaged cells. The absorbance of blanks, determined as a no-enzyme control, was subtracted from each value. The data were normalized to the activity of LDH released from vehicle-treated cells (100 %) and expressed as a percent of the control ± SEM established from *n* = 6 wells per experiment from three to four separate experiments.

#### MTT Reduction Assay

Cell viability assessment was done 24 h (hippocampal cultures) and 48 h (cortical cultures) after treatment with KA. Cell damage was quantified using a tetrazolium salt colorimetric assay with 3-[4,5-dimethyl-thiazol-2-yl]-2,5-diphenyltetrazolium bromide (MTT) as described previously (Jantas et al. 2011). The absorbance of each sample was measured at 570 nm in a 96-well plate-reader (Multiscan, Labsystem). The data after subtraction of blanks (absorbance of cells without MTT) were normalized to the absorbance in the vehicle-treated cells (100 %) and expressed as a percent of the control ± SEM established from *n* = 6 wells per experiment.

#### Immunocytochemistry

In order to morphologically assess the changes in cell viability in cortical and hippocampal cultures after treatment with KA alone, or with KA and ACPT-I (100 or 200 μM), immunostaining was performed using the neuronal marker, anti-MAP-2 according to the procedure described previously (Jantas et al. 2013). At 24 h (hippocampal cultures) or 48 h (cortical ones) after the start of incubation with KA (or without KA in control groups), the cultures were fixed with 4 % paraformaldehyde, permeabilized with PBS containing 0.25 % Triton X-100 for 15 min and incubated for 60 min in blocking solution containing 5 % normal goat serum in PBS-TX-100. Next, cells were incubated with mouse anti-MAP-2 antibody (Santa Cruz, 1:200) for 1 h, washed in PBS, and incubated for the next 60 min in the presence of secondary antibody: anti-mouse Alexa Fluor^®^488 (1:500; Invitrogen, USA). Cells after washing with PBS were mounted with ProLong^®^Gold antifade reagent (Invitrogen, USA), and were examined using a fluorescence AxioObserver.Z1 microscope (Carl Zeiss, Germany) equipped with the software Axiovision 3.1 at excitation wavelengths of 470 nm and images were recorded with a black–white camera (AxioCamMRm, Carl Zeiss).

#### Assessment of Caspase-3 Activity

The assay of caspase-3 activity was determined in samples treated for 6 h with KA either alone or combined with the test compounds according to the method described previously (Domin et al. 2006). After replacing the media with Caspase Assay Buffer (50 mM HEPES, pH 7.4, 100 mM NaCl, 0.1 % CHAPS, 1 mM EDTA, 10 % glycerol, and 10 mM dithiothreitol), the cell lysates (25 μg per sample) were incubated at 37 °C with a colorimetric substrate preferentially cleaved by caspase-3, Ac-DEVD-pNA (*N*-acetyl-asp-glu-val-asp *p*-nitro-anilide). The amounts of p-nitroanilide were monitored by measuring the absorption at 405 nm, continuously over 60 min, using a Multiscan Spectrum Microplate Spectrophotometer (ThermoLabsystems, Vantaa, Finland). In order to confirm the correlation between signal detection and caspase activity, we used Ac-DEVD-CHO (aldehyde substrate; Molecular Probes, USA), which is a specific caspase-3 protease inhibitor. The absorbance of blanks, determined as a no-enzyme control, was subtracted from each value. Data were normalized to the absorbance in vehicle-treated cells, and expressed as a percent of control ± SEM established from *n* = 6 wells per experiment from three to four separate experiments.

#### Data Analysis

The data after normalization as a percentage of control ± SEM were analyzed using GraphPad Prism 4.0 software. One-way analysis of variance (ANOVA) was used to determine the overall significance. The differences between control and experimental groups were assessed with the post hoc Tukey test. The level of significance was determined as *P* < 0.05.

### In Vivo Studies

#### Animals

Male Wistar rats weighing about 250-300 g were used for the experiments. The rats were age-matched and were housed six to a cage on a 12:12 light–dark cycle, with free access to food and tap water. The rats, after cannulae implantation, were housed singly. During the experiment, all efforts were made to minimize animal suffering and to reduce the number of animals used, in accordance with the Local Bioethical Commission Guide for the Care and Use of Laboratory Animals.

#### Cannulae Implantation

The rats were anaesthetized with equithesin and were stereotaxically, bilaterally implanted with chronic guide cannulae aimed at the dorsal hippocampus CA1 region. The guide cannulae (23-gauge stainless steel tubing), secured by dental cement, were anchored to the skull by three stainless steel screws. In order to prevent clogging, stainless steel stylets were placed in the guide cannulae and left until the animals were microinjected.

#### Drug Treatments

Seven days after the cannulae implantation, the rats were unilaterally microinjected with KA (Tocris, USA) into the dorsal hippocampus CA1 region (coordinates: A = +5.7 mm, L = ±2.1 mm, H = +7.2 mm from the interaural line, according to the Paxinos and Watson stereotaxic atlas (Paxinos and Watson 1986). The KA was freshly dissolved in 0.1 M phosphate buffer, pH 7.4, and then microinjected unilaterally in a dose of 2.5 nmol/1 μl. Some rats were additionally injected, through the same cannulae with group III mGlu receptor agonist, ACPT-I (Tocris, USA). The ACPT-I was dissolved in redistilled water before being injected in doses of 7.5 or 15 nmol/1 μl into the CA1 region, 30 min, 1, 3, or 6 h after the initiating KA insult. The contralateral hippocampus of each rat was microinjected with a phosphate buffer and redistilled water, respectively, and used as a control side. The dose of KA was chosen on the basis of our earlier study (Śmiałowska et al. 2003). The doses of ACPT-I were chosen on the basis of previous studies from our laboratory where ACPT-I produced anxiolytic-like effects after intrahippocampal injections (Tatarczyńska et al. 2002; Pałucha et al. 2004).

#### Tissue Preparation and Histology

Seven days after treatment, the rats were killed by an overdose of pentobarbital, their brains were removed, fixed in cold, buffered 4 % paraformaldehyde for 7 days, and then immersed in a buffered 20 % sucrose solution for at least 5 days at 4 °C. The brains were then frozen on dry ice, and 30 μm coronal sections were cut at levels containing the dorsal hippocampus (between bregma −2.12 and −4.30 mm, according to the Paxinos and Watson atlas (Paxinos and Watson 1986). The sections were mounted on glass slides, dried, stained with Cresyl Violet, cover-slipped with Permount, before being used for verification of the injection site and for a histological analysis of the lesion.

#### Stereological Counting of Neurons

The total number of neurons in the pyramidal layer of the CA of the dorsal hippocampus was evaluated by stereological counting. The procedures were performed using a microscope (Leica, DMLB; Leica, Denmark) equipped with a projecting camera and a microscope stage connected to an xyz stepper (PRIOR ProScan) controlled by a computer using Olympus Denmark CAST2 software, as described previously (Domin et al. 2010).

Systemic uniform random sampling was used to choose the sections for the analysis. The first sampling item was randomly taken from the frontal part of the dorsal hippocampus, and all the following sampling items were taken at a fixed distance from the previous one. At least 10–12 sections through the entire length of the dorsal hippocampus were sampled.

The total number of cells (*N*) in the pyramidal layer of the hippocampal CA region was estimated by measuring the reference volume (*V*_ref_ is the area that contains the population of the cells) and the numerical density (*N*_v_) of the cells within the *V*_ref_:

$$N = V_{\text{ref}} \times N_{\text{v}}$$

The pyramidal layer of the dorsal hippocampus CA region was outlined at a lower magnification (5×). CAST2 software provides templates of points in various arrays used in point counting for reference volume estimation. The *V*_ref_ value was determined using point counting methods and applying Cavalieri’s principle (Gundersen and Jensen 1987) according to the formula: *V*_ref_ = ∑pi × *A*(pi) × *t*, where ∑pi is the sum of the number of points (pi) counted, *A*(pi) is the area associated with each point, and *t* is the known distance between sections. The area of the counting frame was *A*(fr) = 3,382 μm^2^.

For determination of the density of cells in the hippocampal CA region, the computer software generated a random selection of sites within the outlined area, from which the density was determined under higher magnification (63×). The cell density (*N*_v_) was estimated using the optical dissector method according to the formula: *N*_v_ = ∑*Q*/∑*P* × *v*(dis), where ∑*Q* is the sum of cells counted from all the dissector frames, ∑*P* is the total number of all the dissector points, and *v*(dis) is the total volume of the dissector.

#### Data Analysis

Statistical analysis was carried out using GraphPad Prism 4.00 software. Differences between the control (contralateral) and KA-lesioned hippocampi (ipsilateral) were compared by a paired two-tailed *t* test. Differences between the KA-lesioned and K+ACPT-I-treated hippocampi were compared by an unpaired two-tailed *t* test. *P* value less than 0.05 was considered statistically significant.

### In Vivo Microdialysis Studies

#### Cannulae Implantation and Treatment with Drugs

Male Wistar rats were anesthetized with ketamine (75 mg/kg i.m.) and xylazine (10 mg/kg i.m.) and placed in a stereotaxic apparatus (David Kopf Instruments, Tujunga, CA, USA). The skull was exposed and small holes were drilled for the insertion of the vertical microdialysis probes in the dorsal hippocampus using the following coordinates: AP = −3.3 mm anterior from the bregma; *L* = +2.2 mm lateral from the sagittal suture; and *H* = −4.0 mm ventral from the dura surface according to the Paxinos and Watson stereotaxic atlas (Paxinos and Watson 1986). The microdialysis probes were constructed as detailed elsewhere (Golembiowska and Dziubina Golembiowska and Dziubina 2004a, 2004b). One day after the surgery and probe implantation, the inlet of the dialysis probes was connected to a syringe pump (BAS, IN, USA) which delivered an artificial cerebrospinal fluid (aCSF) composed of [in mM]: NaCl 147, KCl 4, CaCl_2_ 2.2; pH 7.4 at a flow rate of 1.5 μl/min. After 3 h washing period, when the extracellular level of the neurotransmitters became stable, four baseline samples were collected every 30 min. Next, freshly prepared solutions of KA (50 μM) in aCSF were perfused locally through a microdialysis probe for 30 min. ACPT-I (200 μM) or KA+ACPT-I in aCSF (ACPT-I was administered simultaneously with kainate) were perfused locally through a microdialysis probe for 30 min. Then, the perfusion fluids were switched back to aCSF or ACPT-I for three additional collection periods. The concentration of ACPT-I was chosen on the basis of our in vitro experiments, which have been presented here as being very effective even after 3 h delay. At the end of the experiment, the rats were killed and their brains were examined histologically to validate the correct probe placement.

#### Analytical Procedure

Glutamate was measured in dialysates (20 μl) after derivatization with 4-dimethylaminoazobenzene-4′-sulfonylchloride (DABS-Cl) at 70 °C for 12 min, according to Knecht and Chang (1986). The dabsylated amino acids were separated on an Ultrasphere ODS (4.6 × 150 mm, 3 μm) column (Supelco, Poznań, Poland) by gradient elution, with solvent A (10 mM citric acid, 4 % dimethylformamide) and solvent B (acetonitrile). The dabsylated compounds were detected by measuring an absorbance at 436 nm using the Beckman Amino Acid System Gold with VIS detection.

#### Statistical Analysis

The statistical significance of microdialysis data was calculated using one-way ANOVA for repeated measures, followed by Tukey’s post hoc test. The results were considered statistically significant when *P* < 0.05.

## Results

### The Effect of ACPT-I in Primary Neuronal Cell Cultures

**Fig. 1:**
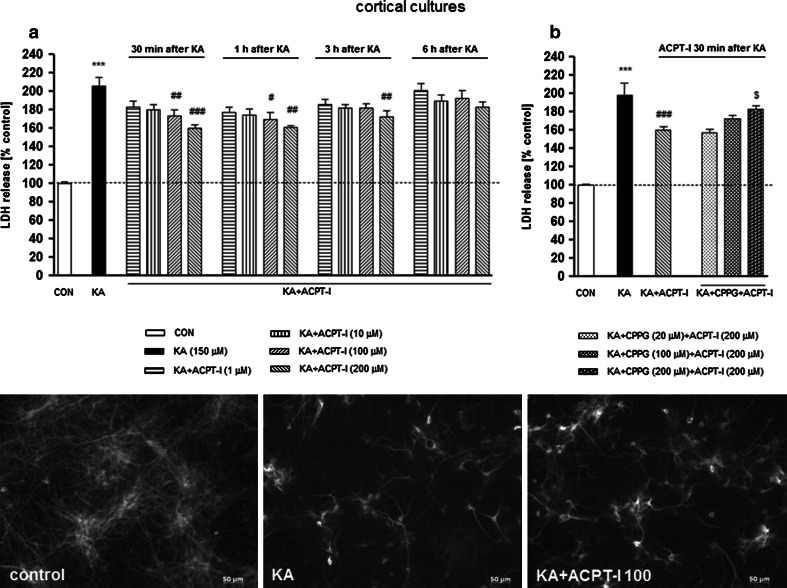
(*upper panel*) **a** The effect of ACPT-I on kainate (KA; 150 μM)-induced LDH release in the primary cultures of a mouse cortical neurons. ACPT-I (1, 10, 100, or 200 μM) was added to the culture medium 30 min, 1, 3, or 6 h after starting the exposure to KA. (*upper panel*) **b** The effect of CPPG on changes in LDH release induced by KA and ACPT-I in primary cultures of a mouse cortical neurons. ACPT-I (200 μM) was added to the culture medium 30 min after KA; CPPG (20, 100, or 200 μM) was applied 10 min before the ACPT-I. LDH was measured 48 h after KA administration. The data were normalized as a percentage of control value and expressed as the mean of *n* ≥ 6 platings ± SEM from 3 to 4 independent experiments. ^***^ *P* < 0.001 (vs. control cultures); ^#^ *P* < 0.05, ^##^ *P* < 0.01, ^###^ *P* < 0.001 (vs. KA-treated cultures); and ^$^ *P* < 0.05 (vs. KA + ACPT-I-treated cultures). (*Bottom panel*) Microphotographs from MAP-2 immunofluorescence of cortical neurons. Numerous clusters of neurons are seen in control cultures. The density of immunostained neurons visibly decreased after 48 h incubation with KA. ACPT-I (100 μM; 1 h after KA) partially prevents the reduction in the number of MAP-2 positive cells in KA-treated cortical neurons. Calibration *bars* 50 μm

**Fig. 2:**
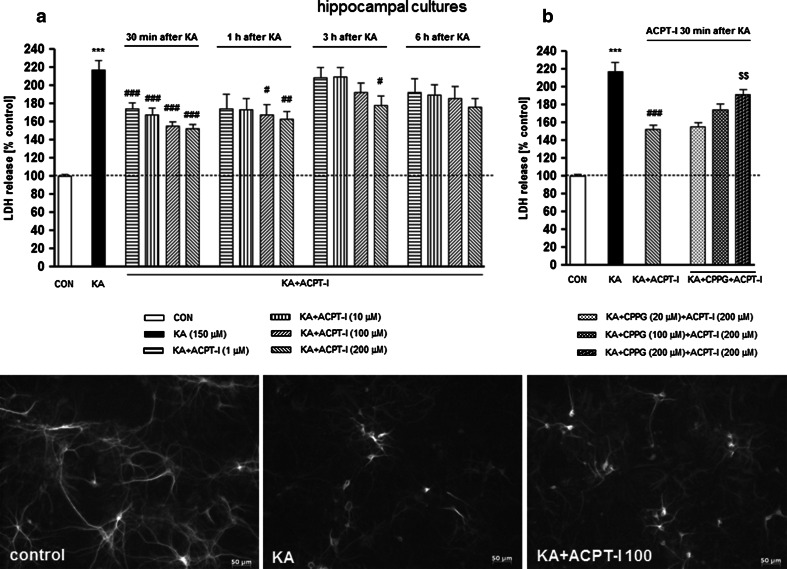
(*upper panel*) **a** The effect of ACPT-I on kainate (KA; 150 μM)-induced LDH release in the primary cultures of a mouse hippocampal neurons. ACPT-I (1, 10, 100, or 200 μM) was added to the culture medium 30 min, 1, 3, or 6 h after starting the exposure to KA. (*upper panel*) **b** The effect of CPPG on changes in LDH release induced by KA and ACPT-I in primary cultures of a mouse hippocampal neurons. ACPT-I (200 μM) was added to the culture medium 30 min after KA; CPPG (20, 100 or 200 μM) was applied 10 min before the ACPT-I. LDH was measured 24 h after KA administration. The data were normalized as a percentage of control value and expressed as the mean of *n* ≥ 6 platings ± SEM from 3 to 4 independent experiments. ^***^ *P* < 0.001 (vs. control cultures); ^#^ *P* < 0.05, ^##^ *P* < 0.01, ^###^ *P* < 0.001 (vs. KA-treated cultures); and ^$^ *P* < 0.01 (vs. KA + ACPT-I-treated cultures). (*Bottom panel*) Microphotographs from MAP-2 immunofluorescence of hippocampal neurons. Numerous neurons with processes are seen in control cultures. The decrease in neurons density and diminution of their processes are found after 24 h incubation with KA. ACPT-I (100 μM; 1 h after KA) partially prevents the reduction in the number of MAP-2 positive cells in KA-treated hippocampal neurons. Calibration *bars* 50 μm

Application of ACPT-I, a group III mGlu receptor agonist, significantly decreased the KA-induced LDH release in both cortical (Fig. 1a, b) and hippocampal (Fig. 2a, b) cell cultures. The effect depended on the ACPT-I concentration, the time of application following exposure to KA and the type of cell culture. In cortical cell cultures, a significant effect in LDH release test was observed when ACPT-I was added at concentrations of 100 or 200 μM at 30 min (about 25–39 % decrease) or 1 h (about 29–38 % decrease) after KA. Significant inhibition was also observed when ACPT-I was applied 3 h after KA, but only at the highest concentration of 200 μM (about 26 % decrease). No effect on LDH release was found when ACPT-I was added 6 h after KA (Fig. 1a). In hippocampal cell cultures, the effect of the group III mGlu receptor agonist was much stronger. When ACPT-I was applied 30 min after KA, a significant inhibition of LDH release was observed not only at its concentrations of 100 or 200 μM (about 57–60 % decrease), but also at 1 or 10 μM (about 39–45 % decrease). When ACPT-I was added 1 h after KA, LDH release was diminished by 45–50 % versus the control value only by concentrations 100 or 200 μM. The protective effect of ACPT-I (200 μM) was also observed with up to a 3 h delay of its application after KA (about 34 % decrease) and no protection was found when ACPT-I was given 6 h after KA (Fig. 2a).

Next, we confirmed the protective effects of ACPT-I found in LDH release assay via assessment of cell viability using biochemical MTT reduction test where ACPT-I significantly increased cell viability after KA treatment both in cortical and hippocampal cell cultures (Table 1). ACPT-I when given alone (1–200 μM) had no effect on viability of cortical and hippocampal neurons measured by LDH release and MTT reduction assays (data not shown). The neuroprotective effects of ACPT-I found in biochemical assays (LDH release and MTT reduction) were confirmed by morphological observation of cortical and hippocampal neuronal cell cultures immunostained with the neuronal marker, anti-MAP-2. It was found that KA (150 μM) applied into the cultures induced a massive neuronal cell death after 24 h in hippocampal cultures or after 48 h in cortical cultures, which was partially prevented by ACPT-I (100 or 200 μM) applied 1 h after KA [Figs. 1, 2 (bottom panels)].

**Table 1:** The effect of ACPT-I (100 or 200 μM) on kainate-induced MTT reduction in eight DIV cortical and hippocampal cultures

	Cell viability (% control ± SEM)
Cortical	
Control	100.00 ± 4.55
KA (150 μM)	62.62 ± 9.06***
KA + ACPT-I (100 μM)	85.37 ± 4.63^#^
KA + ACPT-I (200 μM)	94.80 ± 6.73^##^
Hippocampal	
Control	100.00 ± 6.21
KA (150 μM)	55.27 ± 3.55***
KA + ACPT-I (100 μM)	73.50 ± 6.26^#^
KA + ACPT-I (200 μM)	80.70 ± 3.34^##^

MTT reduction assay was performed after treatment of cells with group III mGluR agonist ACPT-I (100 or 200 μM) 1 h after KA (150 μM) for 48 h (cortical) or 24 h (hippocampal cultures). Data were normalized and expressed as a percentage of control group ± SEM

*** *P* < 0.001 (vs. control cultures); ^##^ *P* < 0.01 and ^###^ *P* < 0.001 (vs. KA-treated cultures)

The strongest neuroprotective effect evoked by ACPT-I at a concentration of 200 μM, given 30 min after KA, was eliminated by the use of the selective group III mGlu receptor antagonist, CPPG. It was found that the application of CPPG, 10 min before ACPT-I, at a concentration of 200 μM, but not 20 or 100 μM, significantly reversed the neuroprotective effect of ACPT-I in both cortical (Fig. 1b) and hippocampal (Fig. 2b) cell cultures. CPPG alone, added to the cell cultures at the same concentrations, had no effect on the LDH level (data not shown).

**Fig. 3:**
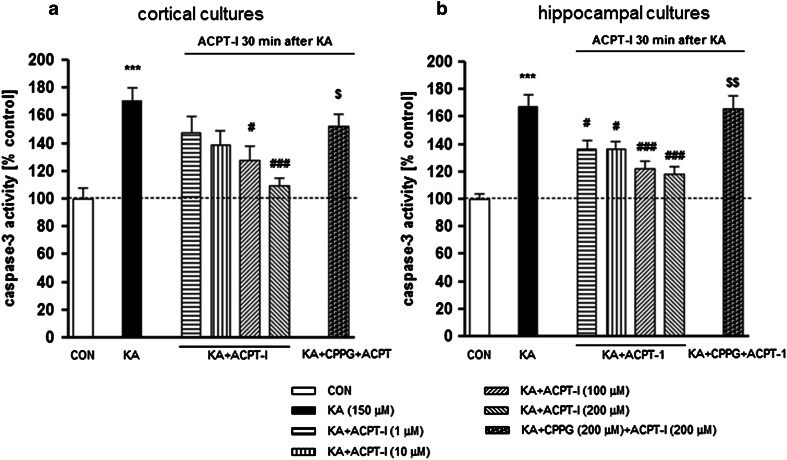
The effect of ACPT-I and CPPG on the KA-induced increase in caspase-3 activity in a mouse’s primary cortical (**a**) and hippocampal (**b**) cultures. Caspase-3 was measured 6 h after starting the exposure to KA. ACPT-I (1, 10, 100, or 200 μM) was added to the culture medium 30 min after KA; CPPG (200 μM) was applied 10 min before the ACPT-I. The data were normalized as a percentage of control value and expressed as the mean of *n* ≥ 6 platings ± SEM from 3 to 4 independent experiments. ^***^ *P* < 0.001 (vs. control cultures); ^#^ *P* < 0.05, ^###^ *P* < 0.001 (vs. KA-treated cultures); and ^$^ *P* < 0.05, ^$^ *P* < 0.01 (vs. KA + ACPT-I-treated cultures)

The biochemical measurement of caspase-3 activity showed a potent activation of this apoptotic enzyme after 6 h of KA intoxication, reaching the value of 170 % of the control in both cortical and hippocampal cell cultures. In cortical neurons, ACPT-I, applied 30 min after KA, significantly inhibited the caspase-3 activity by 43–61 % versus the control value only at its higher (100 or 200 μM) concentrations (Fig. 3a). In hippocampal neurons, it was found that the application of ACPT-I significantly diminished the activity of caspase-3 not only at higher concentrations, 100 or 200 μM (about 48–52 % decrease), but also at lower concentrations, 1 or 10 μM (about 34 % decrease) (Fig. 3b). In both, cortical and hippocampal cultures, inhibition of the caspase-3 activity by ACPT-I (200 μM) was significantly abolished by the group III mGlu receptor antagonist, CPPG, applied 10 min before ACPT-I, at a concentration 200 μM (Fig. 3a, b). Both, ACPT-I and CPPG given alone had no effect on the caspase-3 activity in all the investigated cell cultures (data not shown).

### The Effect ACPT-I After Intrahippocampal Injection In Vivo

**Fig. 4:**
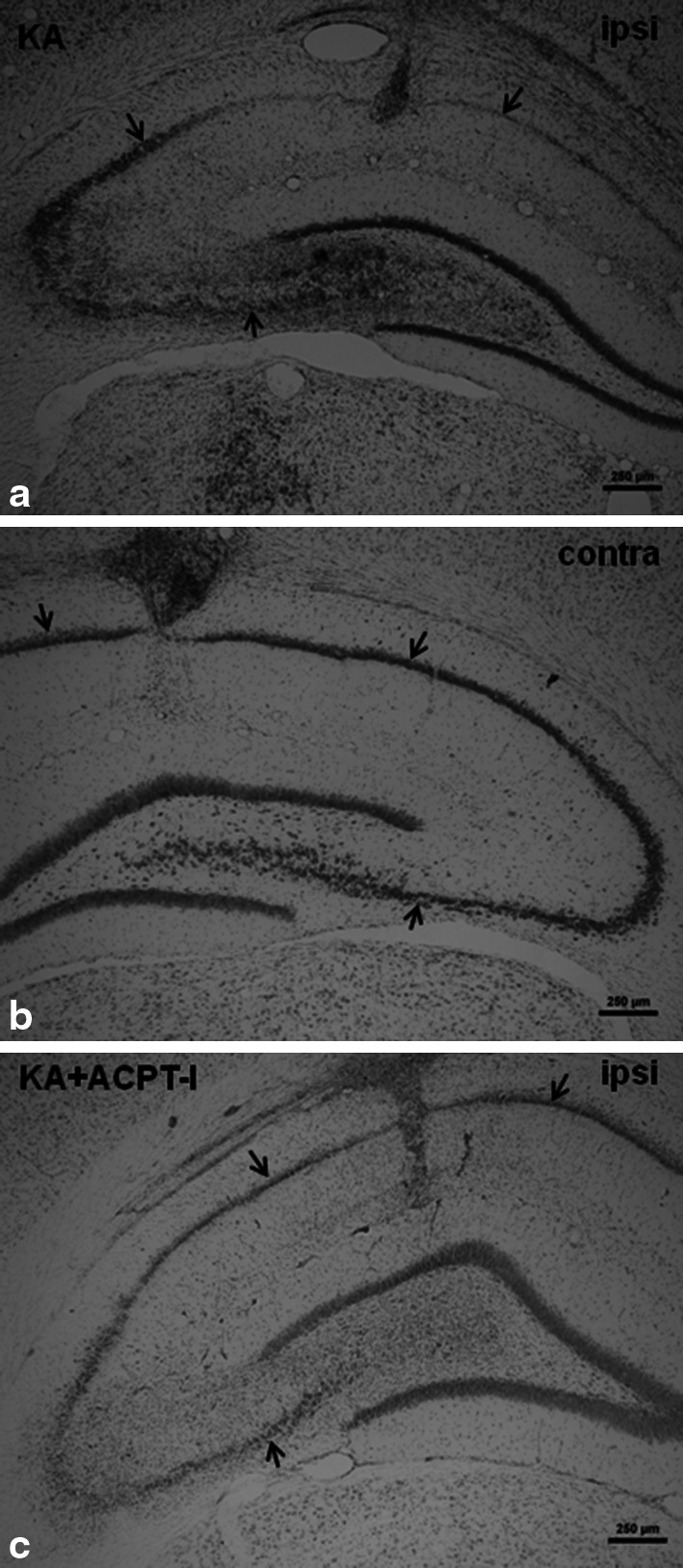
Microphotographs of coronal sections of rat brain hippocampi stained with cresyl violet. *Arrows* indicate a CA pyramidal layer where the neurons were counted. Calibration *bars* 250 μm. **a** Loss of neurons and extensive gliosis can be seen in CA after KA microinjection (2.5 nmol/1 μl) in comparison with the non-degenerated contralateral side (***b***). **c** Neuroprotective effect of ACPT-I (15 nmol/rat) injected into the hippocampus 3 h after KA. The lesion is much smaller than after KA alone

**Fig. 5:**
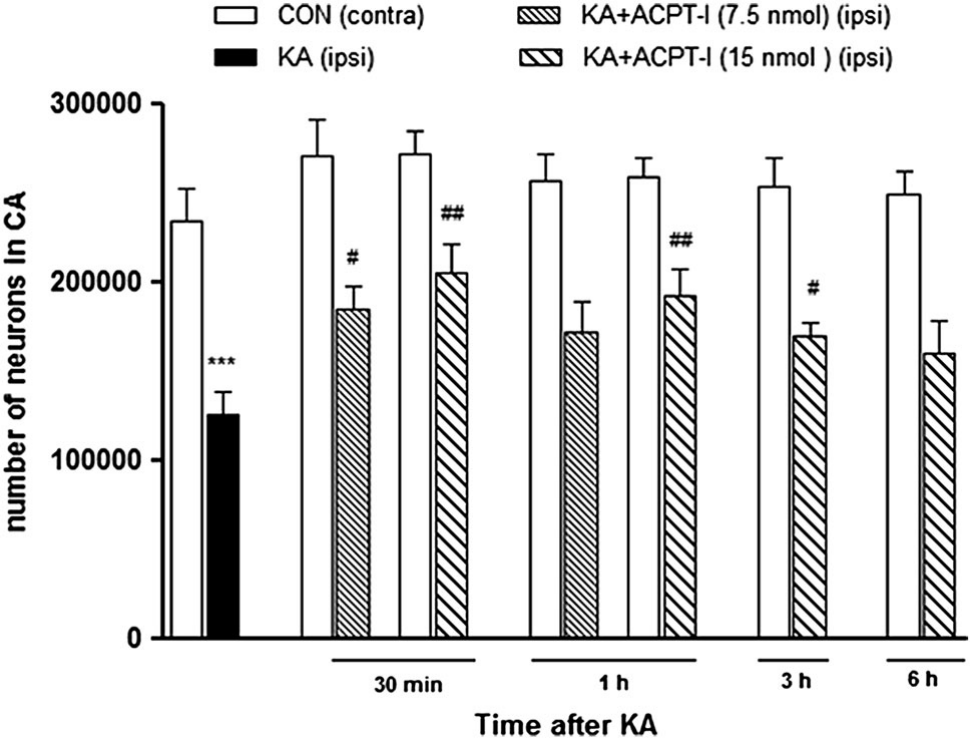
The effect of intrahippocampal injections of KA (2.5 nmol/1 μl) and KA followed by ACPT-I on the number of neurons in the pyramidal layer of CA regions. The results of stereological counting showed neurodegeneration after KA (50 % loss) and neuroprotection induced by ACPT-I given 30 min, 1, or 3 h after KA. No protection was seen when ACPT-I was given 6 h after KA. Each *bar* represents the mean ± SEM of *n* = 6 per group. ^***^ *P* < 0.001 KA (ipsilateral) versus contralateral side, ^#^ *P* < 0.05, ^##^ *P* < 0.01 KA +ACPT-I (ipsilateral) versus KA-lesioned (ipsilateral) hippocampi

KA injected unilaterally at a dose of 2.5 nmol into the CA1 region of the dorsal hippocampus induced an extensive degeneration of neurons in the CA pyramidal layer (Fig. 4a). Stereological counting showed a strong ca. 50 %, reduction in the number of neurons in the pyramidal layer of the ipsilateral dorsal hippocampus in comparison to the contralateral side [*t*(5) = 8.998, *P* = 0.0003; Fig. 5]. Behavioral observations did not show any generalized seizures after KA. Only face twitching and occasionally, movements of the forelimbs were seen.

Cresyl violet staining showed that the extent of lesions in the CA pyramidal layer was significantly smaller in rats treated with the group III mGlu receptor agonist, ACPT-I (Fig. 4c). The effect depended on the dose of ACPT-I and time of its injection following the KA insult. ACPT-I, administered 30 min after KA at doses of 7.5 or 15 nmol per rat, caused a significant increase in the number of living neurons (an increase by 47 and 63 %, respectively) [*t*(8) = 3.077, *P* = 0.0152 and *t*(9) = 3.930, *P* = 0.0035, respectively] in comparison with KA-lesioned hippocampi (Fig. 5).

The neuroprotective effect was also observed when ACPT-I was given 1 or 3 h after the prior KA insult only at a dose of 15 nmol per rat. The results of stereological counting showed a significant increase in the number of living neurons in the CA pyramidal layer of the ipsilateral dorsal hippocampus by 52 and 35 %, respectively, to the time [*t*(10) = 3.317, *P* = 0.0078 and *t*(10) = 2.950, *P* = 0.0145, respectively] in comparison to the side with KA alone (Fig. 5).

Microinjection of ACPT-I 6 h after KA did not induce any protection [*t*(9) = 1.582, *P* = 0.1481; Fig. 5].

### The Effect of ACPT-I on the Extracellular Level of the Glutamate Level in the Rat Hippocampus

**Fig. 6:**
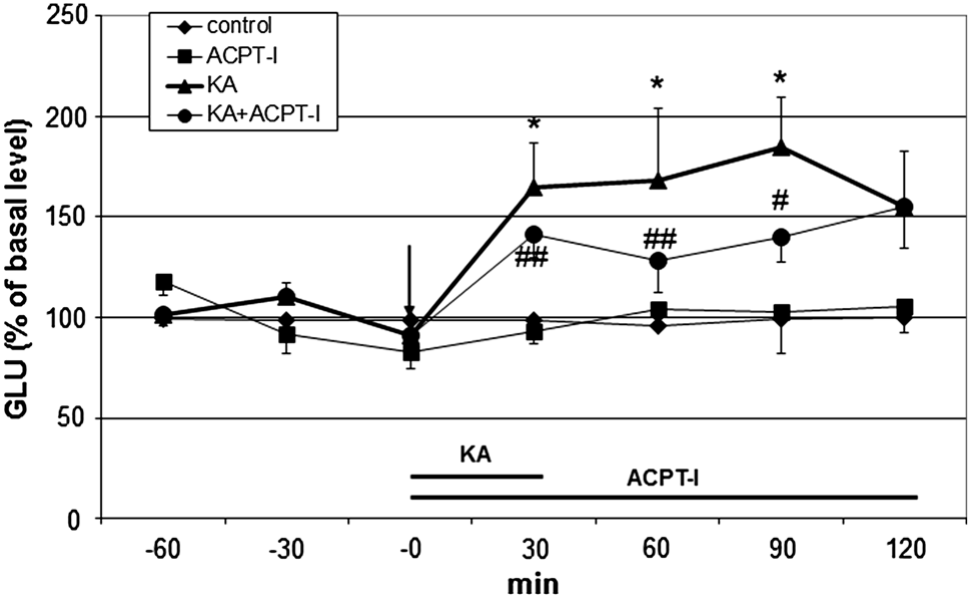
The effect of ACPT-I (200 μM) on the extracellular GLU level induced by kainic acid (KA, 50 μM) in the rat hippocampus. Drug administration is indicated with an *arrow*, while the *horizontal bar* shows the duration of the treatment. The basal extracellular GLU levels (μM) were 0.78 ± 0.08, 0.55 ± 0.06, 1.01 ± 0.06 and 0.84 ± 0.09 in control, ACPT-I, KA and KA + ACPT-I group, respectively. Data are mean ± SEM (*n* = 4–6). Repeated measures of ANOVA and Tukey’s post hoc test. ^*^ *P* < 0.05 versus control; ^#^ *P* < 0.05, ^##^ *P* < 0.01 versus KA-treated group

ACPT-I (200 μM) given into the rat hippocampus did not change the extracellular glutamate (GLU) level at 30, 60, 90, and 120 min after administration (Fig. 6).

KA (50 μM) significantly increased the extracellular GLU level in the rat hippocampus at 30, 60, and 90 min after administration (*P* < 0.05) (Fig. 6). ACPT-I (200 μM), given simultaneously with KA (50 μM), significantly decreased the extracellular GLU level increased by KA at 30, 60, and 90 min after treatment (*P* < 0.05–0.01) (Fig. 6). ANOVA for repeated measures showed a significant effect of the treatment [*F*(3,14) = 18.88, *P* = 0.0003], no significant effect of time [*F*(3,42) = 0.66, *P* = 0.58], and no significant effect of time × treatment [*F*(9,42) = 1.91, *P* = 0.08].

## Discussion

The present results demonstrate that the group III mGlu receptor agonist, ACPT-I, produces neuroprotective effects against kainate-induced excitotoxicity. To the best of our knowledge, this is the first study showing the neuroprotective potential of ACPT-I both in vitro, in primary cultures of mouse cortical and hippocampal neurons, and in vivo after its intrahippocampal injection in the rat. The particularly important finding/observation of our present study was that ACPT-I attenuated the KA-evoked neuronal cell damage after delayed administration (30 min–3 h after KA) in both the in vitro and in vivo studies. Such delayed treatment seems to better correspond to the situation of patients who usually can be treated only some time after injury.

As already mentioned above, so far there have been no studies on the neuroprotective properties of ACPT-I. However, the obtained data in our study are in line with the results demonstrating the neuroprotective effects of other group III mGluR agonists, both in the in vitro and in vivo models of neurodegeneration. Nevertheless, these authors did not investigate the possibility of the delayed application, because in the majority of the studies the compounds were administered predominantly before (Gasparini et al. 1999; Pizzi et al. 2000; Folbergrová et al. 2008; Wang et al. 2012), simultaneously (Bruno et al. 1996; Gasparini et al. 1999; Lafon-Cazal et al. 1999; Bruno et al. 2000; Maj et al. 2003), or shortly after damage (Iacovelli et al. 2002).

Our in vitro results showed that the neuroprotective effect of ACPT-I depended on its concentration, the time of application following exposure to KA, and on the type of cell culture. In hippocampal cell cultures, ACPT-I was more effective than in cortical ones, and a significant neuroprotection was induced by all tested concentrations when the agonist was applied 30 min after the KA. The improved neuroprotective effects of ACPT-I in hippocampal rather than cortical cell cultures may be attributed to the higher density of group III mGlu receptors on glutamatergic neurons and terminals in this structure (Bradley et al. 1996; Shigemoto et al. 1997). Therefore, our in vivo studies concerning the potential neuroprotective effects of ACPT-I have been performed after its intrahippocampal injection.

The in vivo results also showed that the neuroprotective effect of ACPT-I depended on its dose and the time of application following KA. It is worth noting that ACPT-I was still significantly effective when administered as late as 3 h after KA, although that effect was considerably weaker than that observed after a sooner treatment (1 h or 30 min after KA). Therefore, our present data indicate that the neuroprotective activity of ACPT-I is not restricted to the in vitro model, but may also occur in vivo, which could be particularly important for its possible future use in clinical settings. Especially, the effectiveness of the delayed treatment may indicate a potential therapeutic use of similar compounds in patients in whom the neuroprotective treatment can be introduced only a few hours after an injury. The administration of mGlu receptor ligands some time after the induction of neuronal cell damage was also the goal of our previous studies (Domin et al. 2006; 2010; Śmiałowska et al. 2012). We observed that the mGluR5 antagonist MTEP seemed to be particularly promising for neuroprotection, because it prevented excitotoxic neuronal cell damage even when applied 6 h after the toxin in both in vitro and in vivo models (Domin et al. 2006; 2010). In the study by Vernon et al. (2008), it was found that co-administration of the group III mGluR agonist L-AP4 and the group I mGluR5 antagonist MPEP provided an enhanced neuroprotection in the rat in vivo model of Parkinson’s disease. Therefore, the results from our and other laboratories led us to hypothesize that after the co-administration, MTEP and ACPT-I may interact in an additive or synergistic manner resulting in an enhanced neuroprotective effect. However, in our in vitro studies, it was found that simultaneous application of MTEP and ACPT-I did not result in an enhanced neuroprotection compared with treatment with each ligand alone (data not shown).

ACPT-I, a selective group III mGlu receptor agonist, has no activity at other mGluRs (Goudet et al. 2008). In our in vitro study the effect of ACPT-I was inhibited by the group III mGlu receptor antagonist, CPPG, which confirms specificity of the neuroprotective effect of the used agonist via group III mGlu receptors. It is not clear which subtype of group III mGluRs is responsible for neuroprotective effect of ACPT-I. It was established that this agonist was much more potent at mGlu4, 6, and 8, than at mGlu7 receptors (Panatier et al. 2004), and its potency at the mGluR7 subtype was expressed in the millimolar range (Goudet et al. 2008; Stachowicz et al. 2009). The mGlu4, mGlu7, or mGlu8 receptors may, therefore, be responsible for the neuroprotective action of ACPT-I observed in our studies, but not mGlu6 receptors as their expression is limited to the retina (Nakajima et al. 1993). Whether mGluR4, 7, 8 or all of them are involved in the neuroprotective effect of ACPT-I, remains an open question. There are many studies indicating a crucial role of mGluR4 in the protective activity of group III mGluR agonists in cultured mouse cortical cells (Bruno et al. 1995; Gasparini et al. 1999, Bruno et al. 2000). In the study by Lafon-Cazal et al. (1999), it was found that the group III agonist L-AP4 at high concentrations (≥1 mM), necessary for stimulation of mGluR7 (Okamoto et al. 1994; Flor et al. 1997), was neuroprotective against NMDA-induced neuronal death in cultured mouse cerebellar granule neurons. Since L-AP4, with its low micromolar potency, showed a greater affinity for mGlu4, -6, and -8 than for mGlu7 (similar to ACPT-I) (Moldrich et al. 2003), it is, therefore, highly plausible that in our present in vitro study the mGlu4 receptors may be responsible for the neuroprotective effects of ACPT-I, which was effective at concentrations of 100 or 200 μM in mouse cortical neuronal cell cultures.

As already mentioned above, the neuroprotective effect of ACPT-I in vivo has not been tested so far. In our in vivo study, we observed the neuroprotective properties of ACPT-I after intrahippocampal injection in rats. This effect was observed after delayed ACPT-I treatment 30 min to even 3 h after KA injection. The number of living neurons in the CA pyramidal layer significantly increased after ACPT-I in comparison to KA-lesioned hippocampi. Our in vivo findings are in line with several previous animal studies showing the neuroprotective effects of agonists of group III mGluRs against excitotoxic neuronal cell death (Gasparini et al. 1999; Bruno et al. 2000; Folbergrová et al. 2008). The question arises which of the group III mGlu receptors could be involved in the neuroprotective action of ACPT-I in vivo in the hippocampus. Immunohistochemical data have shown that hippocampal glutamatergic neurons and terminals are rich in group III mGlu receptors, especially in mGluR4 and mGluR7, which are both widely distributed in the hippocampus (Bradley et al. 1996; Shigemoto et al. 1997). It has been reported that the mGlu7 receptors are the most widely distributed in the CA regions with mainly presynaptic localization in the neuropil (Shigemoto et al. 1997). The study of Gasparini et al. (1999) revealed that the mGlu7 receptor subtype required high concentrations of the agonist to be activated. It is important to bear in mind that in our present in vivo experiments we used high doses of ACPT-I (7.5 or 15 nmol/rat), thus it may be speculated that just mGlu7 receptors may be responsible for its neuroprotective effect in the CA region of the hippocampus. On the other hand, the role of the mGlu4 receptor subtype in the neuroprotective action of ACPT-I in our model cannot be excluded, as Bruno et al. (2000) postulated that mGlu4 receptors could play a critical role in mediating neuroprotection.

Considering the possible mechanism underlying the neuroprotective action of ACPT-I, it should be noted that the hippocampal formation, especially the CA1 and CA3 regions, was found to contain a high density of KA receptors, hence the pyramidal neurons in the hippocampal CA fields appear to be particularly sensitive to KA-induced neuronal excitation (Coyle 1983; Malva et al. 1998; Nadler et al. 1978). A lot of evidence indicate that the excitotoxicity induced by KA involves the activation of presynaptic KA receptors located on glutamatergic terminals in the hippocampus, thus causing the excessive release of glutamate (Chittajallu et al. 1996; Ferkany and Coyle 1983; Ferkany et al. 1982) and the dysregulation of Ca^2+^ homeostasis (Arundine and Tymianski 2003). We can assume that a possible mechanism of the neuroprotective action of ACPT-I, both in neuronal cultures and in the rat hippocampus, might be related to the reduction of excitatory glutamatergic neurotransmission, as group III receptors are known to inhibit the release of glutamate (Cartmell and Schoepp 2000; Shigemoto et al. 1997). Such inhibition of glutamate release via the activation of group III mGlu autoreceptors was shown, for example, in the CA1 region and dentate gyrus of the hippocampus (Baskys and Malenka 1991; Gereau and Conn 1995; Koerner and Cotman 1981), hippocampal neuronal cell cultures (Forsythe and Clements 1990), and cerebral cortex (Jin and Daw 1998). These receptors are negatively coupled to voltage-gated calcium channels (Conn and Pin 1997) thus, the blockade of calcium entry into the cells, depression of excitatory synaptic transmission and inhibition of glutamate release may cooperate in neuroprotection elicited by agonists of group III mGlu receptors against KA toxicity (Nicoletti et al. 1996). Thus, it seems likely that the neuroprotective effects of ACPT-I in our animal models may be related to the inhibition of glutamate release. In order to verify whether the mechanism underlying the neuroprotective action of ACPT-I may be related to its ability for inhibition of glutamate release, we decided to investigate the possible influence of ACPT-I on KA-induced glutamate release in the rat hippocampus using a microdialysis technique. It was observed in our study that, indeed, ACPT-I significantly diminished the KA-induced increase in glutamate release. It seems to play some role in mediating the neuroprotective effect of ACPT-I. The present data are in agreement with the results of other authors who have shown that group III mGluR agonists such as L-AP4 and ACPT-I caused significant inhibition of glutamatergic and GABAergic transmission in the hypothalamic supraoptic nucleus (Panatier et al. 2004). Since mGlu4 and mGlu7 are densely expressed in the hippocampus (Bradley et al. 1996; Shigemoto et al. 1997), it is not excluded that these subtypes of mGluRs could play predominant role in reducing glutamate release from presynaptic terminals in our microdialysis study.

Since it has been reported that protective activity of the mGluR system against cell injury appears to be linked to the modulation of caspase activity (Maiese et al. 2005), in our in vitro study we examined whether the neuroprotective effect of ACPT-I may be connected with the inhibition of KA-induced caspase-3 activity. There is a strong link between the activation of caspase-3 and apoptotic degradation of genomic DNA. A specific caspase-3 activated DNase has been identified and characterized as an enzyme which is involved in the internucleosomal fragmentation of DNA and, finally, in apoptotic cell death (Sakahira et al. 1998). We observed the maximal induction of caspase-3 activity after 6 h of KA intoxication which was significantly decreased by application of ACPT-I in both cortical and hippocampal cell cultures. In addition, it was found that the effect of the highest concentration of ACPT-I was reversed by CPPG, a potent group III mGluR antagonist. These results strongly indicate the involvement of group III mGlu receptor subtypes in the neuroprotective effect of ACPT-I and we can conclude that there is a direct link between neuroprotection attributed to ACPT-I and inhibition of apoptosis by this compound. The present data are in agreement with the results of other authors who have shown that the activation of group III mGlu receptors by their agonists L-SOP and (R,S)-PPG attenuates the induction of caspases-3, -8, and -9 activities in primary cortical neurons (Zhao et al. 2008). Furthermore, in a study by Wang et al. (2012) the authors observed that the activation of group III mGluRs with L-AP4 attenuated the sevoflurane-induced apoptosis both in vitro in primary hippocampal neuronal cell cultures and in vivo in the rat hippocampus. The authors specifically pointed out that the mGluR7 allosteric agonist AMN082 contributed to the inhibition of sevoflurane-induced apoptosis, whereas the mGluR4 allosteric agonist VU0155041 did not (Wang et al. 2012). In our in vitro study, we did not use the subtype-specific group III mGlu receptor agonists and, thus, we cannot specify which receptors of group III mGluRs are involved in the inhibition of kainate-induced apoptosis by ACPT-I.

In conclusion, the results obtained in this study demonstrated the significant neuroprotective effects of the group III mGlu receptor agonist ACPT-I against excitotoxicity. Neuroprotection evoked by ACPT-I may arise from the inhibition of excitatory glutamatergic transmission. It seems of crucial importance that ACPT-I can diminish excitotoxic neuronal injury even when the treatment is delayed by, from 30 min to 3 h after the toxin. The effectiveness of such late treatment may give us the hope for a potential therapeutic use of similar compounds in patients to whom the neuroprotective treatment can be applied only a few hours after an insult. Therefore, we suppose that group III mGlu receptors may be the promising targets for intervention in a variety of neurodegenerative disorders.
